# Manipulation of autophagy for host-directed tuberculosis therapy

**DOI:** 10.7196/AJTCCM.2019.v25i2.014

**Published:** 2019-07-31

**Authors:** P Gina, M Davids, K Dheda

**Affiliations:** Centre for Lung Infection and Immunity, Division of Pulmonology and UCT Lung Institute, Department of Medicine, University of Cape Town, South Africa

**Keywords:** mycobacterium tuberculosis, autophagy, host directed tuberculosis therapy

## Abstract

*Mycobacterium tuberculosis* (*M. tb*) is one of the world’s most successful human pathogens, infecting ~2 billion people worldwide. Although there are effective drugs against *M. tb*., the disease remains out of control owing to prolonged and toxic treatment. Shorter regimens are urgently required to control TB. Drug-resistant TB (DR-TB) also threatens to derail TB control. These unfulfilled needs could be addressed by the identification and development of host-directed therapeutic agents for TB. Manipulation of the innate immune response, including autophagy, may lead to the identification of cellular pathways that could be exploited to develop host-directed therapeutic interventions. Host-directed therapies (HDTs) aim to augment immune mechanisms against *M. tb* infection and/or reduce excess inflammation, thus preventing end-organ tissue damage, preserving lung function and/or enhancing the effectiveness of TB drug therapy in eliminating infection. HDTs may also have additional advantages for patients with TB/HIV co-infection, as HDTs may reduce the risk of interaction with antiretroviral drugs and the risk of developing immune reconstitution inflammatory syndrome (IRIS) and death. In this review, we discuss the role of autophagy as a potential pathway that could be exploited as a host-directed TB therapeutic agent.

## Background

Approximately one-third of the world’s population is estimated to be latently infected with TB, and are therefore at risk of developing active TB disease during their lifetime.^[1]^ According to the World Health Organization (WHO), in 2017 it was estimated that ~10 million individuals became ill with TB, of whom 9% were HIV-infected (72% in Africa).^[1]^ Drug-resistant tuberculosis (DR-TB) is a major threat to global health.^[1]^ The true burden of DR-TB in Africa is poorly described owing to poor reporting. The African region has the highest DR-TB prevalence: 3.1% of cases in Southern Africa; 2.1% in Central Africa; 1.9% in Western Africa; and 1.7% in Eastern Africa.^[2]^ DR-TB patients endure lengthy and toxic TB treatment but continue to suffer from functional disability due to lung damage from an aberrant host immune response to *M.tb*^[3]^

Research on antimicrobials that directly target *M. tb* must continue but additional approaches are also urgently needed. One of those strategies might involve host-directed therapy (HDT) with FDA-approved compounds targeting manipulation of the innate immune response.^[4]^ Traditionally, *M. tb* drugs are either bacteriostatic (preventing bacterial replication) or bactericidal (directly killing the bacteria). Treatment of drug-susceptible tuberculosis comprises a standard 6-month course of 4 antimicrobial agents, which consists of 2 months of isoniazid, rifampicin, pyrazinamide and ethambutol (2HRZE) and 4 months of isoniazid and rifampicin (4HR).^[5]^ Drug resistance in TB emerges as a result of spontaneous gene mutations in M.tb that render the bacteria resistant to the most commonly used TB drugs.^[6]^ However, HDT compounds act by modulating the host immune response, enabling bacterial killing even at suboptimal drug concentrations, and thus limiting the development of drug resistance.^[7]^ The most prominent cell intrinsic biological pathway targeted by numerous HDT candidates is autophagy within macrophages.^[8]^

HDTs include commonly used drugs for non-communicable diseases with good safety profiles, immune modulatory compounds, biologics and cellular therapies.^[9]^ The clinically relevant examples and progress of these agents as adjunct treatment options for bacterial, viral and parasitic infectious diseases has been reviewed.^[10]^ The examples of HDT agents previously used successfully against pathogens re summarised in Table 1.

TB infection in humans induces a classic inflammatory response. It is the balance between immunopathology and insufficient inflammation that may determine disease severity and outcome. The detrimental effects of inflammation in human hosts are crystallised in the TB immune reconstitution inflammatory syndrome (TB-IRIS).^[11]^ TB-IRIS is paradoxically worsening of TB symptoms with reconstitution of the immune system associated with highly active antiretroviral therapy (HAART).^[11]^ A randomised, controlled trial for adjunctive prednisone showed improvement in symptoms for TB-IRIS.^[12]^ The morbidity associated with inflammatory symptoms of IRIS reflects a general detrimental inflammatory state that can be induced by TB infection. Autophagy has the potential to balance the beneficial and detrimental effects of immunity and inflammation post TB infection.^[13]^

The activation of autophagy by different drugs or compounds may represent a promising treatment strategy against *M. tb* infection and DR-TB. The mediators of autophagy activation include vitamin D receptor signalling, the mechanistic target of rapamycin (mTOR), the AMP-activated protein kinase pathway, sirtuin-1 activation, and nuclear receptors.^[14]^ In the present review, we discuss current knowledge and perspectives on new therapeutic strategies targeting autophagy against TB.

## Autophagy

**Fig. 1:**
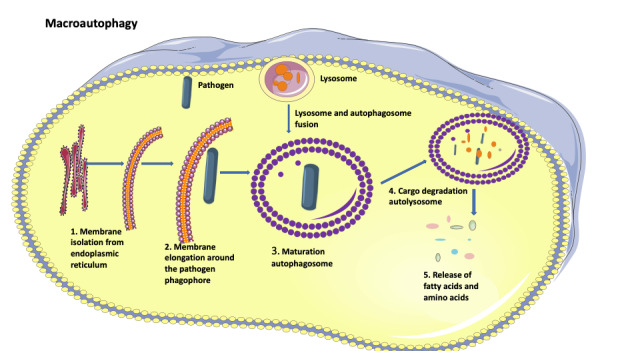
Phases of the autophagy pathway. The autophagic pathway proceeds through several phases, including initiation (formation of a pre-autophagosome structure leading to an isolation membrane, or phagophore), vesicle elongation, autophagosome maturation and cargo sequestration, and autophagosome-lysosome fusion. In the final stage, autophagosome contents are degraded by lysosomal acid hydrolases and the contents of the autolysosome are released for metabolic recycling.

Three distinct types of autophagy have been described: microautophagy, in which the cytosol is directly engulfed by lysosomes;^[15]^ chaperone-mediated autophagy, in which specific proteins are recognised by a cytosolic chaperone and targeted to the lysosome;^[16]^ and macro-autophagy (hereafter referred to as autophagy), in which an isolation membrane, or phagophore, fuses with itself to form an autophagosome with a distinctive double-membrane, which can then fuse with lysosomes (Fig. 1).^[17]^ The autophagy pathway is defined in genetic terms as dependent on autophagy-related (Atg) genes and in morphological terms as the appearance in the cytoplasm of double-membraned organelles termed autophagosomes that capture cytosolic cargo and fuse with lysosome.^[18,19]^ Autophagy is crucial for the maintenance of cellular homeostasis by continuously degrading damaged organelles, long-lived proteins, protein aggregates, and intracellular pathogenic microorganisms.^[20]^

There are two forms of macro-autophagy: non-selective (bulk or generalised), which is autophagic degradation of the cytoplasm usually in response to starvation; and selective autophagy, whereby specific targets in the cytosol are recognised by autophagic receptors and captured by autophagosomes.^[21]^ Xenophagy is a selective autophagy that targets the intracellular microbes for degradation limiting their survival and replication.^[22]^

Several studies have shown that autophagy is associated with different immunological processes in which it: (i) functions as an innate defence mechanism against intracellular microbes, including M.tb, as demonstrated by Gutierrez *et al*.;^[20]^ (ii) is under the control of pattern recognition receptors (PRR), such as toll-like receptors (TLRs), and it acts as one of the immunological output effectors of PRR and TLR signalling (Delgado *et al*.^[23]^ and Xu *et al*. ^[23]^); (iii) is one of the effector functions associated with the immunity-regulated GTPases;^[24]^ and (iv) is activated by Th1 cytokines (which act in defence against intracellular pathogens) and is inhibited by Th2 cytokines (which make cells accessible to intracellular pathogens) (Harris *et al*.).^[25,26]^ The opposing roles of Th1 and Th2 cytokines dictating the ability of macrophages to control intracellular *M.tb* can now be attributed in part to the autophagy activating effect of Th1 cytokines, and autophagy repressing effects of Th2 cytokines.^[27]^

There is growing evidence that autophagy may play a critical role in response to TB. There are studies which demonstrated that Th1 cytokines IFN-γ and TNF-α induce autophagy which enables the macrophages to overcome the phagosome maturation block and inhibit intracellular survival.^[28]^ Conversely, the Th2 cytokines IL-4 and IL-13 inhibit autophagy in murine and human macrophages (Table 2).^[28]^

## Autophagy and *M. tuberculosis*

**Fig. 2:**
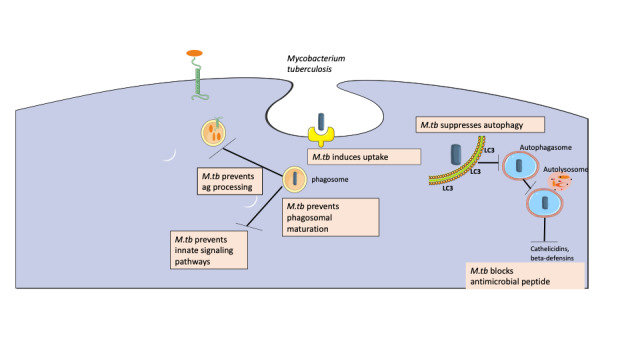
*Mycobacterium tuberculosis* (*M. tb*) persists in the macrophages by subverting multiple intracellular antimicrobial mechanisms. Phagocytosis of *M. tb* is facilitated by various pattern recognition receptors (PRRs); mainly complement receptors (CRs) responsible for uptake of opsonised *M. tb*, mannose receptors (MRs) and scavenger receptors (SR) facilitate uptake of non-opsonised TB bacilli. M.tb prevents antigen (ag) processing and MHCII expression in agpresenting cells. TB pathogen alters the autophagic machinery through the EASAT-6 secretion system-1 (ESX-1) system. The *M. tb*-secreted virulence factors suppress innate immune signalling pathways including autophagy and also block antimicrobial peptides. LC3 = microtubuleassociated protein light chain3

*M. tb* is an intracellular pathogen and thus requires the host cells, i.e. alveolar macrophages, dendritic cells, and neutrophils, for its replication and persistence.^[29]^ Host phagocytic cells provide the synthetic machinery and energy source for *M. tb*, and also possess intrinsic defence mechanisms that are triggered by infection.^[30]^ Therefore, *M. tb* must possess strategies to block such defences as shown in Fig. 2.^[31]^ By modifying the host defence mechanisms, *M. tb* is able to persist and survive in resting macrophages.^[32]^ *M. tb* persists in the macrophages by subverting multiple intracellular antimicrobial mechanisms. The major virulence feature of pathogenic mycobacteria rests on the ability to parasitise the host’s scavenger cells, especially macrophages. After phagocytic uptake by macrophages, *M. tb* is not delivered to phagolysosomes for degradation, which is the hallmark of autophagy. Instead, it continues to reside within the phagosome which is prevented from maturing or fusing with lysosomes. In this manner, live pathogenic *M. tb* remain in a weakly acidified environment away from the hostile environment of phagolysosomes. M.tb is retained in the phagosome where it will survive or replicate within the macrophages. A variety of mechanisms have been suggested that contribute to the survival of *M. tb* within the macrophages, including inhibition of phagosome-lysosome fusion, inhibition of the acidification of phagosomes, resistance to killing by reactivated oxygen and nitrogen intermediates and modification of the lipid composition of *M.tb* cell membrane.^[33]^

Although several mycobacterial factors have been implicated in immune evasion, the mechanisms and specific mediators involved remain unknown. Vergne *et al*. ^[34]^ described how *M. tb* lipoarabinomannan (LAM) causes phagosome maturation arrest by interfering with intracellular signalling and membrane trafficking. LAM from virulent *M. tb* blocks cytosolic calcium by preventing the interaction PI3kinase hVPS34 with calmodulin, which is crucial for autophagosome maturation.^[34]^ Romagnoli et al. ^[35]^ demonstrated that virulent strains of *M. tb* impair late steps of autophagy by secreting ESX-1, which inhibits autophagosome-lysosome fusion.

**Fig. 3:**
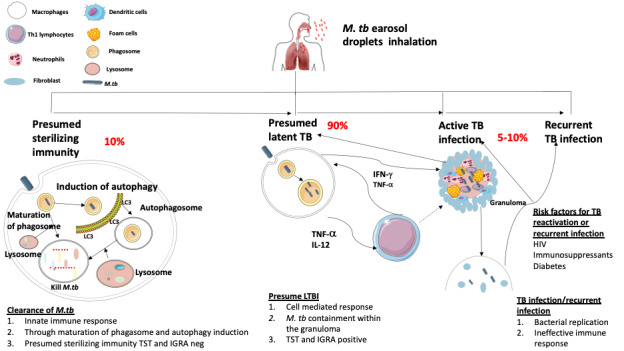
The spectrum of *Mycobacterium tuberculosis* infection. Mycobacteria are inhaled into the lung alveoli. Here the infection may be cleared by presumed sterilising innate immune response (these mechanisms may determine the results of immunodiagnostics tests such as the tuberculin skin test (TST) and interferon-gamma release assay (IGRA). In the remainder, the infection may progress to latent tuberculosis infection (LTBI) or in a small percentage to active tuberculosis infection.

It has become clear that *M. tb* is capable of inhibiting autophagy, thus allowing it to replicate within macrophages (Figs2 and 3).^[34]^ Macrophage activation by Th1 cells and their cytokines, IFN-γ and TNF-α, improves *M. tb* control but this activation was shown to be insufficient to completely clear the infection.^[36]^ Active TB emerges either as progressive primary TB infection or as a consequence of immune suppression after long stages of pathogen persistence (Fig. 3).^[37]^

## Autophagy induction as a host-directed TB therapeutic option

HDT is an emerging concept in the treatment of *M. tb* where host immune response is modulated to achieve better control of TB.^[13]^ HDT can interfere with host mechanisms that are required by a pathogen for productive replication or persistence.^[38]^ Alternatively, HDT can enhance the immune response by stimulating mechanisms that are involved in host defence against the pathogen, target pathways that are disrupted by a pathogen, contribute to hyper-inflammation, and modulate host factors that lead to dysregulation responses at the site of pathology.^[39]^

Indeed, several HDT approaches rely on the repurposing of licensed drugs for other diseases, such as cancer, metabolic and cardiovascular diseases.^[40]^ Therefore, the concept of HDT for *M. tb* infection is novel and provides untapped opportunities that are urgently needed in the face of increasing DR-TB infection.^[39]^ Nevertheless, most HDT approaches are not considered to be stand-alone therapies but are combined with existing TB drugs.^[3]^ Some studies have shown that induction of autophagy might be achievable by treatment with metformin (MET) and/or nitazoxanide, which can promote *M. tb* kill.^[13,32]^ Targeting autophagy could lead to effective treatments for DR-TB, shorter treatments for drug sensitive tuberculosis, and more adjunctive therapy using FDA-approved drugs such as metformin, nitazoxanide, statins, vitamin D and imatinib compounds (Table 3).^[4]^ Several drugs with potential for repurposing such as TB HDTs already have well-defined safety and pharmacokinetic profiles and are ready to progress to randomised, controlled clinical trials that will evaluate their effectiveness in TB, TB–HIV co-infection and TB with other diseases.^[40]^ Vitamin D induces the expression and release of innate antimicrobial peptides such as cathelicidin, promoting autophagosome maturation and TB killing.^[41]^ The diabetes drug metformin enhances macrophage autophagy by promoting phagolysosome fusion. Metformin achieves this by increasing mitochondrial production of reactive oxygen species, and also induces expression of AMP-activated protein kinase, a potent inducer of autophagy.^[13]^ Statins such as simvastatin and rosuvastatin have anti-inflammatory effects, and induce autophagy and phagosome maturation.^[51]^ The anticancer kinase inhibitor imatinib interferes with *M. tb* entry and intracellular survival in host cells and may help to clear *M. tb* by promoting autophagy.^[52]^

## Conclusion

*M. tb* is an intracellular pathogen that alters the ability of the host’s phagocytic cells to clear the infection. Manipulation of the innate immune response should contribute to intracellular *M. tb* killing. ^[31]^ The activation of autophagy by diverse compounds may represent a promising treatment strategy against M. tb infection, including drug-resistant strains. ^[42]^ Important mediators of autophagy, including vitamin D receptor signalling and the AMP-activated protein kinase pathway, are of great importance in identifying compounds which can be used as HDT. ^[43]^ Understanding the mechanisms and key players involved in modulating antibacterial autophagy will provide innovative improvements in anti-TB therapy via an autophagy-targeting approach.^[44]^

Thus, the identification of novel compounds and pharmacological host targets (in immune pathways) that could amplify and facilitate effective host immune responses to help eliminate TB bacilli is an attractive approach.^[14]^ However, this newly emerging treatment option should not be misinterpreted as an exclusive alternative; rather it should be seen as a synergistic addon to anti-TB drugs.

Exploitation of already licensed FDA-approved drugs, despite their current indication, may have therapeutic properties against TB, and the evaluation of these compounds is more cost-effective than the development of new drugs. Undoubtedly, future treatment regimens for *M. tb* will converge on the concept of personalised medicine, providing the best possible combinations that are adjusted, not only for the pathogen, but also for the patient. However, the clinical benefit versus the higher costs of these approaches remain to be determined. Moreover, HDTs as an adjunct strategy for the treatment of *M. tb* is still in its infancy and requires further investigation.

## Figures and Tables

**Table 1:** Host-directed therapeutic agents for infectious disease

**Pathogen and** **type of agent**	**Examples of host-** **directed therapy**	**Mechanism of action**	**Developmental stage**
Bacterial infections			
*Mycobacterium tuberculosis* repurposed drug^[13]^	Metformin	Modulation of inflammation and activation of intracellular antimicrobial defences.	Preclinical
*Streptococcus pneumoniae* antibiotic^[45]^	Azithromycin, erythromycin	Reduces local tissue inflammation through anti-inflammatory activities in communityacquired pneumonia.	In clinical use (though clinically relevant anti-inflammatory effect is controversial)
Viral infections			
Hepatitis C cytokine therapy^[46]^	Pegylated interferon α and β	Potentiation of pro-inflammatory antiviral immune response.	In clinical use
HIV repurposed drug^[47]^	Valproic acid	Reactivation of latent HIV infection and making new viral progeny susceptible to ART and immune attack by enhancing gene transcription.	Preclinical
Parasitic disease			
Malaria repurposed drugs^[48]^	Desferriomxamine	Ferrochelatase inhibitor reduces *Plasmodium spp.* burden in erythrocytes.	Preclinical
Leishmaniasis repurposed drug^[49]^	Imiquimod, resiquimod	TLR agonist that induces B-cell activation and pro-inflammatory cytokine signalling.	In clinical use

**Table 2:** Known modulatory effects of cytokines on autophagy

**Cytokine/chemokine**	**Effect on autophagy**
IFN-γ^[20,25]^	Induces autophagy in human and murine macrophages: Dependent on *IRGM* genes.
TNF-α^[28,50]^	Induces autophagosome formation in human and murine macrophages.
IL-1α and IL-1β^[50]^	Autophagy regulates the secretion of IL-1β and IL-1α in antigen-presenting cells. This is one of the mechanisms in which autophagy regulates the inflammatory response in antigen-presenting cells.
IL-4 and IL-13^[26]^	Inhibits starvation-induced autophagy via activation of the Akt pathway, which activates mTOR. However, IL-4 did not influence rapamycin-induced autophagy because it acts directly on the mTOR, thereby bypassing Akt signalling.

**Table 3:** Mechanisms of different compounds in autophagy induction

**Compounds**	**Mechanisms**
AMPK pathway	
Metformin^[13]^	Metformin inhibits *M. tb* growth by activating AMPK-mediated autophagy pathway in macrophages, which promotes phagolysosome fusion. Additionally, metformin selectively induces production of mitochondrial reactive oxygen species (mROS). In mice infected with *M. tb*, metformin improves pulmonary pathology and reduces bacterial load.
Small molecules/chemicals	
Simvastatin^[51]^	Inhibitors of 3-hydroxy-3-methylglutaryl-coenzyme A reductase and agonists of peroxisome proliferator activated receptor-γ. Statins reduce the formation of lipid droplets by mycobacteria and reduce survival of *M. tb* in macrophages by inducing autophagy and maturation of the phagosome.
Imatinib^[52]^	Direct pharmacological effect on macrophage function, promoting acidification and maturation of phagosomes. In mouse models, it reduces intracellular *M. tb* survival *in vitro*. It also increases neutrophil and monocyte numbers, contributing to anti-TB host immune response.

*M. tb* = *Mycobacterium tuberculosis*

AMPK = adenosine monophosphate-activated protein kinase

mTOR1 = mammalian target of rapamycin

IL1R = interleukin-1 recepror

TB = tuberculosis
